# Influence of preoperative corticosteroid treatment on rate of diagnostic surgeries in primary central nervous system lymphoma: a multicenter retrospective study

**DOI:** 10.1186/s12885-021-08515-y

**Published:** 2021-06-29

**Authors:** Florian Scheichel, Franz Marhold, Daniel Pinggera, Barbara Kiesel, Tobias Rossmann, Branko Popadic, Adelheid Woehrer, Michael Weber, Melitta Kitzwoegerer, Klaus Geissler, Astrid Dopita, Stefan Oberndorfer, Wolfgang Pfisterer, Christian F. Freyschlag, Georg Widhalm, Karl Ungersboeck, Karl Roessler

**Affiliations:** 1 Karl Landsteiner University of Health Sciences, Krems, Austria; 2 Department of Neurosurgery, University Hospital St. Poelten, Dunant-Platz 1, 3100 St. Poelten, Austria; 3 Department of Neurosurgery, Medical University of Innsbruck, Innsbruck, Austria; 4 Department of Neurosurgery, Medical University Vienna, Vienna, Austria; 5 Department of Neurosurgery, Donauspital SMZ-Ost, Vienna, Austria; 6 Department of Neurosurgery, Neuromed Campus, Kepler University Hospital, Johannes Kepler University, Linz, Austria; 7 Institute of Neurology, Medical University Vienna, Vienna, Austria; 8 Department of Research Management, Karl Landsteiner University of Health Sciences, Krems, Austria; 9 Department of Pathology, University Hospital St. Poelten, St.Poelten, Austria; 10 Sigmund Freud Private University, Vienna, Austria; 11 Institute for Pathology and Microbiology, Donauspital SMZ-Ost, Vienna, Austria; 12 Department of Neurology, University Hospital St. Poelten, St.Poelten, Austria

**Keywords:** Primary central nervous system lymphoma, Corticosteroid therapy, Diagnostic rate

## Abstract

**Background:**

Corticosteroid therapy (CST) prior to biopsy may hinder histopathological diagnosis in primary central nervous system lymphoma (PCNSL). Therefore, preoperative CST in patients with suspected PCNSL should be avoided if clinically possible. The aim of this study was thus to analyze the difference in the rate of diagnostic surgeries in PCNSL patients with and without preoperative CST.

**Methods:**

A multicenter retrospective study including all immunocompetent patients diagnosed with PCNSL between 1/2004 and 9/2018 at four neurosurgical centers in Austria was conducted and the results were compared to literature.

**Results:**

A total of 143 patients were included in this study. All patients showed visible contrast enhancement on preoperative MRI. There was no statistically significant difference in the rate of diagnostic surgeries with and without preoperative CST with 97.1% (68/70) and 97.3% (71/73), respectively (*p* = 1.0). Tapering and pause of CST did not influence the diagnostic rate. Including our study, there are 788 PCNSL patients described in literature with an odds ratio for inconclusive surgeries after CST of 3.3 (CI 1.7–6.4).

**Conclusions:**

Preoperative CST should be avoided as it seems to diminish the diagnostic rate of biopsy in PCNSL patients. Yet, if CST has been administered preoperatively and there is still a contrast enhancing lesion to target for biopsy, surgeons should try to keep the diagnostic delay to a minimum as the likelihood for acquiring diagnostic tissue seems sufficiently high.

## Background

**Fig. 1:**
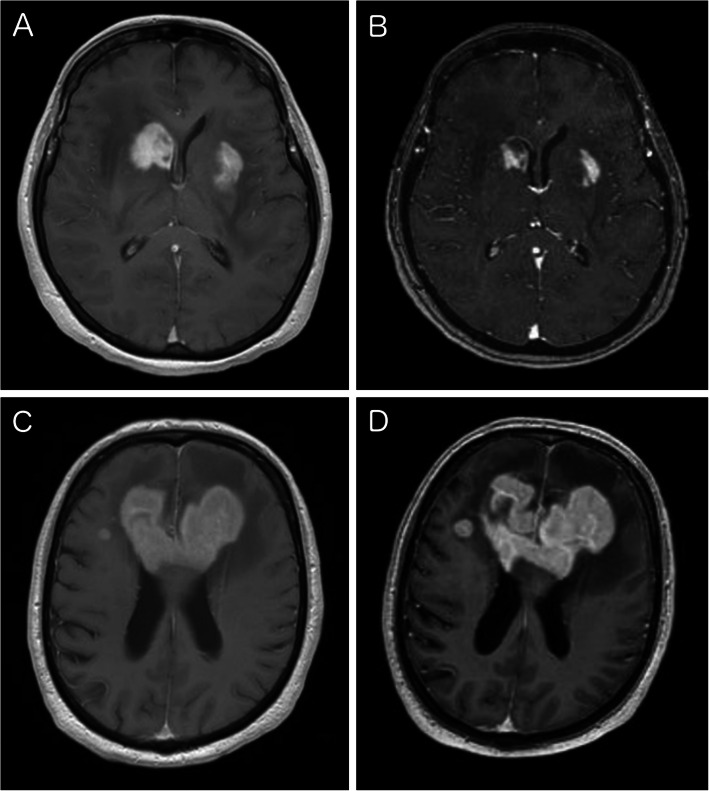
Typical MRI of PCNSL. Preoperative MRI showing multifocal contrast enhancing lesions on T1-weighted sequences (**a**). The patient received 12 mg dexamethasone per day for 8 days in a peripheral hospital and regression was visible on navigational MRI (**b).** CST was then paused 2 days prior to surgery. MRI of a patient with a large bifrontal lesion affecting the corpus callosum (**c**). The patient received 12 mg dexamethasone per day for 3 days following first MRI. The CST was paused immediately after transfer of the patient. Second MRI was performed after 4 days off of CST showing distinct progression (**d**)

Primary central nervous system lymphoma (PCNSL) is a rare disease and accounts for approximately 3% of all intracranial tumors [1]. Cerebrospinal fluid cytology combined with typical radiological appearance can in some cases obviate the need for surgery [2]. However, biopsy is necessary for pathological confirmation of the diagnosis in most patients [3, 4]. PCNSL cells are potentially highly sensitive to corticosteroid therapy (CST) and may react with cell arrest, apoptosis and transient shrinkage of the tumor mass caused by induction of the p38-MAPK pathway [5–7] (Fig. 1). Accordingly, CST may lead to morphological changes in up to 52% of cases which may hinder histopathological diagnosis [8, 9]. Therefore, if clinically possible, CST should be avoided preoperatively until biopsy has been performed [2]. However, preoperative CST is often administered initially due to the patients’ presentation with neurological symptoms, prior to consultation of a neurooncological center.

In day-to-day practice, there is no generally accepted standard procedure for such cases and protocols differ immensely between centers. If clinically possible, recent guidelines advocate tapering of CST in case of a radiological remission and to defer biopsy until new progression in closely performed follow-up MRI [2, 10]. This could lead to a significant delay of diagnosis and definitive treatment with uncertain implications on the prognosis. On the other hand, recent reports showed that the impact of preoperative CST on the diagnostic yield of biopsy may not be as high as formerly described [11–16].

The aim of the present multicenter retrospective study was thus to perform a comprehensive analysis on the influence of preoperative CST on the rate of diagnostic surgeries in PCNSL considering dose, duration and timing of pause of CST. Furthermore, we compared our results to the available literature to estimate the risk for inconclusive surgeries after preoperative CST.

## Methods

In the present study all patients that were diagnosed with an intracranial PCNSL between January 2004 and September 2018 at four neurosurgical centers in Austria (University Hospital St. Poelten - Karl Landsteiner University of Health Sciences; Medical University Vienna; Medical University of Innsbruck; Donauspital SMZ-Ost – Vienna) were retrospectively analyzed. Approval of the institutional review board and local ethics committee of every participating center was obtained.

Each center performed a database query to find all patients that were diagnosed with a cranial PCNSL during the study period.

We excluded immunocompromised patients and patients with systemic lymphoma manifestation in staging examinations who were subsequently diagnosed with secondary CNS lymphoma. Furthermore, patients with unclear preoperative corticosteroid status were ruled out.

### Corticosteroid therapy

Detailed information about preoperative CST was collected from patient records. For calculation of the cumulative dose of CST, loading dose and daily doses were summed up and converted to dexamethasone equivalent.

### Rate of diagnostic surgeries

All patients included in this study were diagnosed with a PCNSL and treated based on this diagnosis. The percentage of patients with need for repeat surgery to ensure diagnosis was calculated and compared.

### Volumetric analysis of contrast enhancement

For each patient a volumetric analysis of all contrast enhancing lesions as well as the single lesion that was targeted for biopsy was performed using Brainlab i-plan©. Tumor lesions were therefore marked on preoperative T1-weighted navigational MRI sequences with contrast agent. The lesion that was targeted for biopsy was defined by surgery report and postoperative cranial CT.

### Comparison to literature

A literature analysis was performed to find studies on rate of diagnostic surgeries in corticosteroid pretreated PCNSL patients. We included studies with at least 10 patients that were of consecutive design and investigated diagnostic rates in both patients with CST and without.

### Statistical analysis

Statistical analyses were performed using IBM SPSS Statistics for windows version 26 (IBM Corp.). Given normal distribution (tested by Kologorow Smirnow test) metric data are described using mean and standard deviation. Skewed data are summarized using median and range. Categorical data are presented as absolute frequencies and percentages.

To test for differences in the diagnostic yield in PCNSL patients with and without preoperative CST Fisher-Exact test was assessed.

A *p*-value < 0.05 was considered to indicate statistically significant results.

## Results

A total of 160 patients were diagnosed with PCNSL in the study period. After exclusion of 14 immunocompromised patients and 3 immunocompetent patients with missing data on preoperative CST status, 143 patients were included in the final analysis.

### Patient characteristics

The median age of our patient cohort was 67.7 years (range 21.2–86.2 years). The study cohort was evenly distributed with 71 (49.7%) females and 72 (50.3%) males. Almost all patients were diagnosed with a B-cell-lymphoma (141 cases, 98.6%) and 2 patients with a T-cell lymphoma (1.4%). All patients showed contrast enhancing lesions in preoperative imaging. Table 1 shows demographic, surgical and radiological data.

**Table 1:** Demographic, surgical and radiological data

Parameter	With preoperative CST	Without preoperative CST	***p***-value
Patients	70 (49%)	73 (51%)	
Female	39 (55.7%)	32 (43.8%)	0.182
Male	31 (44.3%)	41 (56.2%)	
Age	64.9 +/− 12.1	65.8 +/− 11.4	0.625
Location			0.090
Periventricular	10 (14.3%)	21 (28.8%)	
Frontal	15 (21.4%)	9 (12.3%)	
Temporal	6 (8.6%)	7 (9.6%)	
Parietal	4 (5.7%)	6 (8.2%)	
Occipital	1 (1.4%)	1 (1.4%)	
Cerebellar	1 (1.4%)	6 (8.2%)	
Other and multilobular	33 (47.1%)	23 (31.5%)	
One lesion	31 (44.3%)	29 (39.7%)	0.614
Multifocal	39 (55.7%)	44 (60.3%)	
Volume of the targeted lesion	8.6 ccm (0.1–64.1)	7.8 ccm (0.2–61.9)	0.500
Total volume of all intracranial lesions	9.7 ccm (0.1–64.1)	11.4 ccm (0.6–76.3)	0.344
Histological diagnosis			0.497
B-cell lymphoma	70 (100%)	71 (97.3%)	
T-cell lymphoma	0 (0%)	2 (2.7%)	
Multiple surgeries necessary			1.0
No	68 (97.1%)	71 (97.3%)	
Yes	2 (2.9%)	2 (2.7%)	
Type of surgery			0.160
Stereotactic biopsy	46 (65.7%)	59 (80.8%)	
Open biopsy	14 (20.0%)	7 (9.6%)	
Resection	9 (12.9%)	6 (8.2%)	
Endoscopic biopsy	1 (1.4%)	1 (1.4%)	

### Corticosteroid treatment

Preoperative CST was administered in 70 patients (49%) whereof 38 patients continued their CST until surgery (26.6%). CST was paused up to 7 days in 13 cases (9.1%) and more than 7 days in 15 cases (10.5%). In four patients with paused preoperative CST there was uncertainty on timing of the pause. The remaining 73 cases did not receive any preoperative CST (51%).

Median preoperative pause of CST in the 28 patients with paused CST was 8.5 days (range 1–58). Exact duration of preoperative CST could be determined in 57 of 70 patients and showed a median duration of 8 days (range 1–339). In the vast majority of cases dexamethasone was the active substance (66 cases, 94.3%) followed by methylprednisolone (4 cases, 5.7%). The median maximal dose per day in dexamethasone-equivalent was 14 mg (range 8–40 mg, *n* = 60) and the median cumulative dose was 128 mg (range 8–560 mg, *n* = 53).

### Corticosteroid treatment and rate of diagnostic surgeries

Two patients out of 70 (2.9%) that received preoperative CST and two out of 73 (2.7%) without preoperative CST needed a second biopsy to ensure the diagnosis (*p* = 1). Table 2 shows detailed information about histology, surgery and corticosteroid therapy of the four cases with non-diagnostic first surgery.

**Table 2:** Detailed analysis of patients needing a second surgery

Patient nr.	Corticosteroid therapy prior to first surgery	Type of first surgery	Histopathologic result	Corticosteroid therapy after first surgery	Type of second surgery	Histopathological result
Patient 1	Ongoing preoperative dexamethasone therapy over 26 days, maximal dose per day 12 mg, cumulative dose 236 mg	Stereotactic biopsy	Diffuse inflammation without evidence of lymphoma cells	Reduction of corticosteroid therapy, but no discontinuation. Patient was on 1 mg dexamethasone per day at time of second surgery	Stereotactic biopsy, 6 months after first surgery	Diffuse large B-cell lymphoma
Patient 2	No preoperative corticosteroid therapy	Stereotactic biopsy	Brain parenchyma without evidence of tumor	Contradictorily information in documentation whether corticosteroid therapy was tapered in between surgeries	Resection, 1 month after first surgery	Diffuse large B-cell lymphoma
Patient 3	Preoperative dexamethasone therapy for 3 days with a total dose of 52 mg and a max. daily dose of 24 mg. Corticosteroid therapy was paused 7 days prior to surgery	Stereotactic biopsy	T-cell mediated CD3 and CD8 positive inflammation with few perivascular and intra-parenchymatous lytic B-cells; unspecific result	Postoperative corticosteroid therapy was reconvened and paused again 7 days prior to second surgery	Open biopsy 18 days after first surgery	Diffuse large B-cell lymphoma
Patient 4	No preoperative corticosteroid therapy	Stereotactic biopsy	Unspecific inflammation with infiltration of B- and T-cells	No data on corticosteroid therapy prior to second surgery available	Stereotactic biopsy 2 months after first surgery	Diffuse large B-cell lymphoma

Preoperative pause of CST did not influence the result with inconclusive surgeries in 2.6% with ongoing CST (1 of 37 cases) and 2.9% without or with paused CST (3 of 102 cases) respectively (*p* = 0.942).

There was no statistically significant relation between the duration of CST and the rate of diagnostic surgeries (*p* = 0.803). Patients without CST, CST of less than 7 days and CST of more than 7 days had an inconclusive surgery in 2.7% (2 of 73 cases), 4.8% (1 of 21 cases) and 2.8% (1 of 36 cases), respectively.

### Type of surgery

All four patients that needed a second surgery to secure the diagnosis had a non-diagnostic stereotactic biopsy before (3.8%, 4 out of 105 cases). Tissue gained with remaining surgical strategies (38 cases, consisting of resection, open biopsy and endoscopic biopsy) allowed the diagnosis of PCNSL in 100% but there was no statistically significant difference (*p* = 0.573).

Histological analysis in non-diagnostic surgeries showed diffuse inflammation in three cases and normal brain parenchyma in one case (Table 2).

### Volumetric analysis of contrast enhancement

Volumetric analysis could be performed in 134 patients. In 8 patients the navigational MRI was not available for analysis and in one patient the contrast enhancement was so diffuse that no clear border could be defined. Median total volume of all intracranial contrast enhancing lesions was 9.7 ccm (range 0.1–64.1, *n* = 64) compared to 11.4 ccm (range 0.6–76.3, *n* = 70) in patients with and without preoperative CST (*p* = 0.344), respectively. The single lesion targeted for biopsy showed a median volume of 8.6 ccm (range 0.1–64.1) in patients after preoperative CST and 7.8 ccm (range 0.2–61.9) in patients without (*p* = 0.500). Unfortunately, volumetric analysis could only be performed in one out of four patients that needed a second surgery. In this patient with preoperative CST a lesion with a volume of 5.8 ccm was targeted and total volume of all lesions was 33.8 ccm. In one patient, without CST, volumetric analysis was not possible due to diffuse blurry contrast enhancement. In the remaining two cases the navigational MRI was not available for analysis. However, in one of these patients, with preoperative CST, the radiological report described multiple spherical contrast enhancing lesions with a diameter of 8 to 9 mm. In the other case, without CST, two contrast enhancing spherical lesions with a diameter of 2.5 cm each and multiple smaller ones were described.

A comparison between preoperative volumes before and after administering of CST was considered. In 64 patients with preoperative CST there were multiple preoperative MRIs, yet only the navigational MRI had a proper slice thickness that allowed reasonable volumetric analysis. Analysis of structural MRI changes showed a regression in 15 patients (23.4%), no dynamic in 12 patients (18.8%) and a progression despite CST in 37 patients (57.8%). No patient showed complete vanishing of the contrast enhancing tumor. Median time from navigational MRI to surgery in patients with CST was 2.5 days. The two patients that needed a second surgery after CST showed either no change or a progression in their MRIs after CST.

### Comparison to literature

Literature analysis yielded 8 studies (including the present one) that describe rates for diagnostic surgeries in patients with and without preoperative CST (Table 3). These studies describe a total of 788 patients. Inconclusive biopsies were observed in 10.3% after preoperative CST (48 out of 465 cases) and in 3.4% without (11 out of 323 cases, *p* < 0.001). Therefore, the odds ratio for an inconclusive biopsy after preoperative CST was 3.3 (CI 1.7–6.4).

**Table 3:** List of studies on rate of diagnostic surgeries after preoperative CST with study populations of at least 10 PCNSL patients

Study	n	Study period	Patients with CST	Inconclusive biopsies	Patients without CST	Inconclusive biopsies	***p***-value ^c^
Haldorsen et al. [17]	45	1989–1998	36	8 (22%)	9	0 (0%)	nsf
Porter et al. [11]	107	1985–2005	68	8 (12%)	39	5 (13%)	nsf
Manoj et al. [13]	72	1991–2010	26	5 (19%)	46	0 (0%)	0.005
Shaw et al. [12]^, a^	67	2000–2010	57	4 (7%)	10	0 (0%)	nsf
Binnahil et al. [14]^, b^	20	2007–2013	15	0 (0%)	5	0 (0%)	–
Velasco et al. [16]	280	2005–2014	175	20 (11%)	105	4 (4%)	0.045
present study	143	2004–2018	70	2 (3%)	73	2 (3%)	nsf
Bullis et al. [15]	54	2009–2018	18	1 (6%)	36	0 (0%)	nsf
Total	788		465	48 (10.3%)	323	11 (3.4%)	< 0.001

^a^ only cerebral cases of PCNSL were included; ^b^ one case of low-grade B-cell lymphoma was excluded, ^c^ *p*-values were calculated by using a chi-square test

Of all 788 patients, 497 were described in studies that treated the majority of their patients after 2005. Older studies described inconclusive surgeries for patients with CST and without CST in 13.4% (25 out of 162) and 4.8% (5 out of 99, *p* = 0.021), respectively, with an odds ratio of 3.1 (1.1–8.2). More recent studies showed inconclusive biopsies in 8.3% with CST and 2.7% without (*p* = 0.09) with an odds ratio of 3.2 (CI 1.3–8.0).

In 302 patients the type of surgery could be determined with certainty. Stereotactic biopsy was performed in 234 patients and showed a rate of inconclusive surgeries with preoperative CST and without of 8.6 and 1.9% respectively (*p* = 0.026). Open surgical strategies in the remaining 68 patients yielded a histological diagnosis in all cases.

In 263 patients it was documented if CST had been preoperatively paused. There was no detectable statistically significant difference on rate of inconclusive surgeries with 9.4% (17 out 180) in patients with ongoing CST and 7.2% in patients with paused CST (6 out of 83). Median pause, if documented, was either 8.5 days (*n* = 28, range 1–58, current study; NB: exact duration of preoperative pause could not be determined in 4 patients) or 15 days (*n* = 51, IQR 4–42, Velasco et al.) [16].

## Discussion

### Preoperative CST and rate of diagnostic surgeries

Preoperative CST can lead to an impairment of diagnosis in PCNSL patients ranging from complicating histopathological diagnosis to complete disappearance of lymphoma cells [8, 9]. However, we found no statistically significant decrease in the rate of diagnostic surgeries in our study cohort with and without preoperative CST with 97.1 and 97.3% respectively (*p* = 1.0). This study is among one of the largest concerning preoperative CST in PCNSL. Yet, as there are possibly only narrow differences in diagnostic rates, our patient number was not sufficiently high to be able to state with certainty that the diagnostic rates are alike regardless of preoperative CST status. Therefore, we compared our results to the existing literature on influence of preoperative CST on PCNSL. Up to date there are 8 consecutively designed studies (including the present one) that describe rates of diagnostic surgeries for patients with and without preoperative CST and thereby produce conflicting results (Table 3) [11–17]. Altogether they describe 788 patients with cerebral PCNSL of whom 59% received preoperative CST and 41% did not. Patients with preoperative CST had an inconclusive surgery in 10.3% in comparison to 3.4% without CST (*p* < 0.001). The odds ratio for an unsuccessful biopsy after preoperative CST was thus 3.3 (CI 1.6 to 6.4).

Interestingly, rates of inconclusive biopsies were lower in studies with the majority of patients treated after 2005 what might be explained by improved surgical, radiological and pathological techniques. Older studies described inconclusive surgeries for patients with CST and without CST in 13.4 and 4.8%, respectively. More recent studies showed inconclusive biopsies in 8.3% with CST and 2.7% without. However, the odds ratio for an inconclusive biopsy after CST remained about the same with 3.1 (CI 1.1–8.2) before 2005 and 3.2 (1.3–8.0) thereafter.

As these are heterogeneous studies, these findings need to be interpreted with caution. Information on dose, duration and timing of pause of preoperative CST might be crucial factors when interpreting these results. Tapering and pause of preoperative CST as well as preoperative duration of CST did not show any influence on rate of diagnostic surgeries in our study population, though this could be explained by the low overall number of inconclusive biopsies. In the literature, preoperative tapering and pause of CST did not seem to have an influence either with rates of inconclusive surgeries of 9.4% with ongoing CST and 7.2% with paused CST, respectively. However, the case number is too low to state this with certainty and the duration of pause could not be analyzed with the available data. Consequently, there is no clear evidence what amount of time is necessary to overcome the potential negative effect of temporary preoperative CST on the diagnostic rate in PCNSL. Surgeons therefore have to weigh up the risk of an inconclusive biopsy against the delay of surgery, diagnosis and subsequently therapy.

### Type of surgery

All patients in our study population who needed a second surgery to secure diagnosis had undergone stereotactic biopsy as first surgery resulting in a diagnostic rate for stereotactic biopsy of 96.2%. This is comparable to the diagnostic yield of stereotactic biopsy of different brain tumors, that has been described within the range of 91–96% in literature [18, 19]. The remaining surgical strategies (open biopsy, resection and endoscopic biopsy) were able to gain diagnostic tissue in 100%. The difference was not statistically significant as stereotactic biopsies were by far the most frequently performed procedure (*p* = 0.573).

It is noteworthy that there was a higher percentage of open biopsies (20% vs 9.6%, Table 1) and resections (12.9% vs 8.2%) in patients after CST. As these strategies yield more tissue for diagnosis, this could have diminished the rate of inconclusive surgeries in patients after CST.

Two patients who needed repeat surgery in our study cohort underwent another stereotactic biopsy that was then successful. However, the remaining two patients underwent either an open biopsy or resection the second time (Table 2). The surgical strategy was probably changed hoping that gaining more tissue increases the chance for a diagnosis.

The role of neurosurgery in PCNSL patients had been limited to gain tissue for histopathological analysis for years as resection did not show a survival benefit in older studies [4]. Recently, Weller et al. described an improvement in progression free survival in PCNSL patients with subtotal or gross total resection [3]. Yet, in this study patients with a single lesion underwent resection more frequent than patients with multiple lesions which might have biased the outcome. Contrary to these findings, Houillier et al. did not find a survival benefit for resection in multivariate analysis in their recently published study of 1002 PCNSL patients [20]. Therefore, this topic has to be addressed in further studies and until then stereotactic biopsy remains the first choice in suspected PCNSL. However, safety of open surgery in PCNSL improved [21] and it holds the benefit of acquiring more tissue for histopathological analysis. This could be helpful in challenging cases as there could be intratumoral heterogeneity especially in regard to response on CST. In patients who need repeat surgery and show an accessible lesion, open biopsy or resection might therefore be considered. The decision to perform open surgery in suspected PCNSL still has to be made on individual basis and the possible benefit has to be strictly calculated against potentially higher peri-procedural risks and morbidity.

In the literature there are 302 patients described in studies where the authors differed between stereotactic biopsy and other types of surgery that potentially yield more tissue for analysis like open biopsy or resection. In 234 stereotactic biopsies the rate of inconclusive results was 8.6% (11 out of 128 cases) with CST and 1.9% (2 out of 106 cases) without (*p* = 0.026) while all 68 cases (28 without CST, 40 with CST) with open surgical techniques yielded diagnostic tissue.

### Radiological and volumetric analysis

Distinct regression of PCNSL after CST has been described in up to 40% and gave PCSNL the name “ghost/vanishing tumor” [22]. While this phenomenon has been assumed diagnostic for PCNSL formerly, it has also been described for other brain tumors and is accounted as obsolete nowadays [23–26].

**Fig. 2:**
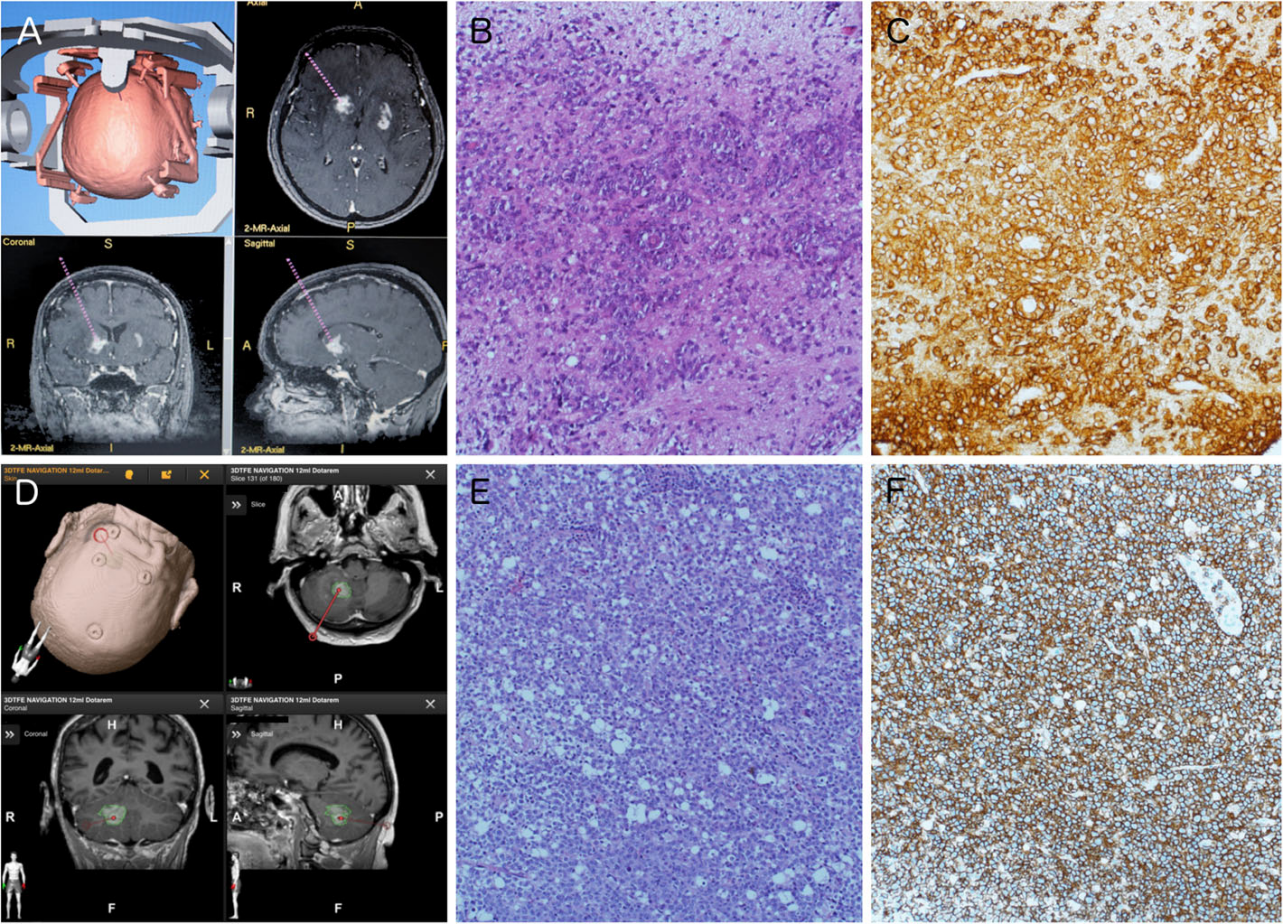
Exemplary histopathological microphotographs. Navigational MRI (**a**) showing the target point for stereotactic biopsy in the same patient as in Fig. 1, A and B. Despite preoperative CST and radiological regression, the histopathological analysis was clearly diagnostic for PCNSL showing typical large pleomorphic lymphoblastic tumor cells with patternless growth, partly cuffed around vessels and few apoptotic tumor cells (**b**). Immunohistochemistry was excessively positive for CD20 (**c**). Navigational MRI and target point (**d**) of a patient without preoperative CST. Microphotographs show diffusely patternless growth of lymphoblastic tumor cells in HE staining (**e**) and distinct positive immunohistochemistry for CD20 (**f**) in this patient without preoperative CST. All histopathological images were generated with 200-fold magnification. Recognizable color differences are due to different age of histopathological specimens

Recent guidelines advice to defer surgery if there was a radiological remission of PCNSL after CST [2, 10]. However, these guidelines do not specify a standard what to consider a significant regression after CST. Established response criteria for PCNSL refer to definitive therapy and not CST alone and furthermore they are not applicable for lesions before histological diagnosis [27]. Therefore, it is an individual decision what extent of regression is considered problematic. Distinct regression may be easily assessed as illustrated in Fig. 1, but a slightly different layering of the MRI might falsify the estimation in certain cases. Additionally, the relative response of a larger lesion might be more obvious than in a small one. Patients with multifocal PCNSL might also show regression of one lesion and progression of another. Radiological regression is often used as a surrogate parameter for response to CST and the diagnostic yield of surgery in clinical practice. However, to the best of our knowledge, no study showed a clear correlation between radiological response and diagnostic yield. No patient in this study showed complete remission of contrast enhancement after CST and despite regression in some cases, there was still a contrast enhancing lesion left on navigational MRI, that could be targeted for surgery or biopsy in all patients (Fig. 2). There was no statistically significant difference in the median volume of the targeted lesion between patients after preoperative CST with 8.6 ccm compared to 7.8 ccm without CST. In absence of cases with complete vanishing of the tumor, our findings can only be applied to lesions with visible contrast enhancement even after CST. Furthermore, size and localization of the lesion, as well as its response to CST have to be taken into account in surgical decision making. A lesion that shows a distinct shrinkage or almost vanished in serial preoperative MRIs after CST may be considered suggestive for an increased risk of non-diagnostic biopsy. Accordingly, a visible remaining contrast enhancing lesion after CST in immediate preoperative imaging seems to be a crucial indicator for a promising diagnostic yield in PCNSL.

### Limitations

1. Patients who underwent an inconclusive surgery are usually closely followed by serial imaging and repeat surgery. However, we cannot preclude that few patients with PCNSL may have died before or refused repeat surgery. These patients were therefore not diagnosed with PCNSL at lifetime and are missing in our study population. We recognize that this constitutes a major limitation.
2. Histopathological analysis was performed at each hospital and tissue samples were sent to a reference center if diagnosis was questionable. A central histopathological review was therefore performed in 55.2% of cases and 3.5% were reviewed in other reference centers. Three of the four patients with inconclusive biopsies were centrally reviewed. In the fourth patient there was no doubt that the tissue samples showed no tumor and therefore no second opinion was obtained. The lack of consecutive central histological review is considered a major limitation of this study. Furthermore, volume of tissue samples was not routinely assessed in all patients and could therefore not be analyzed.
3. There were relatively more open biopsies/resections and less stereotactic biopsies in the group with preoperative CST. This may obscure the potential negative effect of preoperative CST on diagnosis in PCNSL and should be considered when interpreting our results.

## Conclusion

Preoperative CST should be avoided if PCNSL is suspected as the rate of inconclusive surgeries could be increased. However, if CST has already been established, the necessary extent and effect of pausing CST is questionable and the probability for a successful biopsy appears sufficiently high. When deciding to perform biopsy after preoperative CST, the size and volume of the lesion, its location and its potential response to CST need to be considered to diminish the probability for an inconclusive biopsy.

## Data Availability

The data that support the findings of this study are available on reasonable request from the corresponding author Franz Marhold. The data are not publicly available due to them containing information that could compromise patient privacy. Data regarding the comparison to literature was collected from the cited published articles [11–17].

## Abbreviations

PCNSL: Primary central nervous system lymphoma
CST: Corticosteroid therapy
